# Sarin-Induced Neuroinflammation in Mouse Brain Is Attenuated by the Caspase Inhibitor Q-VD-OPh[Fn fn4]

**DOI:** 10.1124/jpet.123.001820

**Published:** 2024-02

**Authors:** Ekta J. Shah, William C. Grunwald, Teresa L. Garrett, Thomas L. Brown, David R. Cool

**Affiliations:** Departments of Pharmacology and Toxicology (E.J.S., W.C.G, T.L.G., D.R.C) and Neuroscience, Cell Biology and Physiology (T.L.G., T.L.B.), Wright State University, Dayton, Ohio

## Abstract

Organophosphates cause hyperstimulation of the central nervous system, leading to extended seizures, convulsions, and brain damage. Sarin is a highly toxic organophosphate nerve agent that has been employed in several terrorist attacks. The prolonged toxicity of sarin may be enhanced by the neuroinflammatory response initiated by the inflammasome, caspase involvement, and generation/release of proinflammatory cytokines. Since neurodegeneration and neuroinflammation are prevalent in sarin-exposed animals, we were interested in evaluating the capacity of quinolyl-valyl-O-methylaspartyl-[-2,6-difluorophenoxy]-methyl ketone (Q-VD-OPh), a pan caspase inhibitor to attenuate neuroinflammation following sarin exposure. To test this hypothesis, sarin-exposed C57BL/6 mice were treated with Q-VD-OPh or negative control quinolyl-valyl-O-methylglutamyl-[-2,6-difluorophenoxy]-methyl ketone, sacrificed at 2- and 14-day time points, followed by removal of the amygdala and hippocampus. A Bio-Rad 23-Plex cytokine analysis was completed on each tissue. The results suggest that exposure to sarin induced a dramatic increase in interleukin-1*β* and 6 other cytokines and a decrease in 2 of the 23 cytokines at 2 days in the amygdala compared with controls. Q-VD-OPh attenuated these changes at the 2-day time point. At 14 days, six of these cytokines were still significantly different from controls. Hippocampus was less affected at both time points. Diazepam, a neuroprotective drug against nerve agents, caused an increase in several cytokines but did not have a synergistic effect with Q-VD-OPh. Treatment of sarin exposure with apoptosis inhibitors appears to be a worthwhile approach for further testing as a comprehensive counteragent against organophosphate exposure.

**SIGNIFICANCE STATEMENT:**

A pan inhibitor of caspases (Q-VD-OPh) was proposed as a potential antidote for sarin-induced neuroinflammation by reducing the level of inflammation via inflammasome caspase inhibition. Q-VD-OPh added at 30 minutes post-sarin exposure attenuated the inflammatory response of a number of cytokines and chemokines in the amygdala and hippocampus, two brain regions sensitive to organophosphate exposure. Apoptotic marker reduction at 2 and 14 days further supports further testing of inhibitors of apoptosis as a means to lessen extended organophosphate toxicity in the brain.

## Introduction

Sarin is a highly toxic organophosphate (OP) nerve agent that was first produced for chemical warfare in 1937 and has been used as a terrorist and chemical weapon ever since. Within 30 to 40 minutes sarin acts as an irreversible inhibitor of the primary target, acetylcholinesterase (AChE), in both the peripheral and central nervous systems (Munro, 1994; Chauhan et al., 2008; Moshiri et al., 2012). Standard of care for OP-toxicity includes a combination of atropine to help relieve symptoms of ACh accumulation, pralidoxime that targets reactivation of AChE enzyme activity, and diazepam that acts as an anticonvulsant to suppress seizures caused by the OP (Augerson, 1998; Wiener and Hoffman, 2004). None of these, however, were designed or are able to address a prolonged response following the original OP exposure (Casida and Quistad 2004).

Specific problems after OP exposure are that OPs cause convulsions/seizures that cannot be eliminated by the standard of care, which then allows for prolonged and increased activation of microglia and astrocytes, which are responsible for releasing proinflammatory cytokines, e.g., interleukin (IL)-1*α*, IL-1*β*, tumor necrosis factor (TNF)-*α*, and prostaglandin E2 (PGE2) (McDonough and Shih 1993; Majno and Joris, 1995; Chapman et al., 2006; Krysko et al., 2008). Proinflammatory cytokines are also a part of the “inflammasome that is driven by caspase 1, which includes nuclear targeting of nuclear factor kappa-light-chain-enhancer of activated B cells to promotor elements that drive the production of IL-1*β*, IL-6, IL-18, and TNF-*α* (Zhang et al., 2016). The end result is a cascade of released cytokines, chemokines, enzymes, and other factors that orchestrate neurodegeneration (Ramesh et al., 2013). Inhibition of caspase 1 has been shown previously to reduce neurodegeneration in cell culture models, leading to the premise that it is a candidate target for drug development that could alleviate multiple neurodegenerative diseases (Pan et al., 2016). Thus, inhibition of caspases is a new avenue to explore in new drug design for counter agents of OP exposure.

**Fig. 1.**
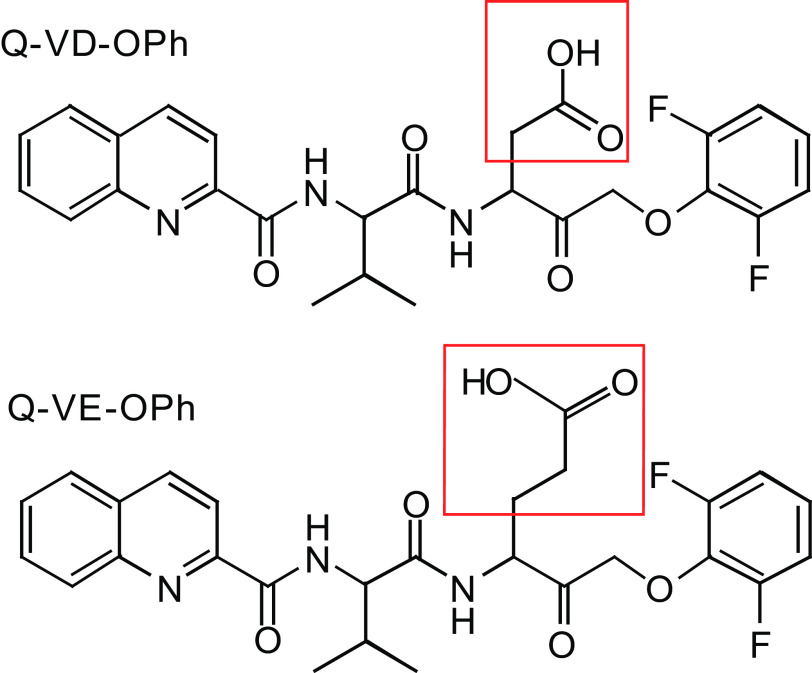
Diagram showing chemical structures of Q-VD-OPh and Q-VE-OPh. The structures were generated with CS ChemDraw (Cambridge Software) and a red box drawn in Adobe Illustrator CC to indicate the position of the aspartic acid (Q-VD-OPh) or glutamic acid residue (Q-VE-OPh). The mass and purity of the compounds were analyzed by IonTrap mass spectrometry (Bruker HCTUltra) and the results reported elsewhere (Southerland et al., 2010).

The present study was designed to test the caspase inhibitor quinolyl-valyl-O-methylaspartyl-[-2,6-difluorophenoxy]-methyl ketone (Q-VD-OPh) for its effects on neuroinflammation caused by the OP sarin. Q-VD-OPh has been shown to act as a neuroprotectant in a neonatal rat stroke mode and to reduce chemical toxicities, e.g., 1-methyl-4-phenyl-1,2,3,6-tetrahydropyridine, malonate, and 3-nitropropionic acid (Antar et al., 2009; Pan et al., 2016). Q-VD-OPh (Fig. 1) permeates cell membranes including the blood-brain barrier and blocks the activation of caspases, the cleavage of specific substrates, and the formation of caspase-specific DNA ladders (Caserta et al., 2003; Yang et al., 2004). Q-VD-OPh is proposed to form an irreversible thioether bond between its aspartic acid moiety and the cysteine active site of the caspase enzyme, although the exact mechanism has not been completely elucidated (Pan et al., 2016). Q-VD-OPh has also been reported to act as an anti-inflammatory, potentially acting through a caspase-activated process (Melnikov et al., 2002). In addition to Q-VD-OPh, a structurally similar control compound is also available wherein a glutamate residue is substituted for the aspartate residue in Q-VD-OPh. Quinolyl-valyl-O-methylglutamyl-[-2,6-difluorophenoxy]-methyl ketone (Q-VE-OPh) has much diminished caspase inhibition activity compared with Q-VD-OPh (Fig. 1) (Southerland et al., 2010).

In the present study, our goals were to determine the extent to which sarin would elicit an inflammatory response in specific brain regions, i.e., amygdala and hippocampus, and to test the capacity of the caspase inhibitor, Q-VD-OPh, to reduce the inflammatory response in these tissues. As a negative control, we synthesized Q-VE-OPh, wherein a glutamate was substituted for the aspartate residue (Southerland et al., 2010). Furthermore, it was of interest to determine whether the combination of diazepam and Q-VD-OPh would have an additive effect on neuroinflammation. The results of the experiments presented here suggest that sarin does induce a larger inflammatory response than what has been reported in the past, e.g., IL-1 *β*, IL6, TNF-*α*, and PGE2 (Chapman et al., 2006; Garrett et al., 2013). In addition, we show that Q-VD-OPh does provide some protection from the sarin-induced neuroinflammation in the amygdala and hippocampus. Finally, we show that the presence of diazepam does cause an increased inflammatory response that is not entirely diminished by the addition of Q-VD-OPh.

## Materials and Methods

### Sarin, 2-(O-cresyl)-4h-1:3:2-Benzodioxaphosphorin-2-Oxide, Q-VD-OPh, Q-VE-OPh.

For this study, sarin (isopropyl methylphosphono-fluoridate) was synthesized at MRI Global, verified by their chemists, and kept as a dilute solution in isopropyl alcohol. All dilutions were in 0.9% saline. Q-VD-OPh and Q-VE-OPh were both synthesized by T.L.B. at Apoptrol, LLC and validated by IonTrap mass spectrometry by D.R.C. (Southerland et al., 2010) (see conflict of interest statement). The 2-(o-cresyl)-4H-1:3:2-benzodioxaphosphorin-2-oxide (CBDP) was a generous gift from Dr. D.M. Maxwell. All other drugs and chemicals were of liquid chromatography/mass spectrometry grade.

### Animals.

The mice used in this study were adult female C57BL/6 mice from Harlan Laboratories, 8 to 12 weeks old, weighing between 20 and 28 g. Mice used as controls were housed and treated at the Wright State University animal facility under an approved IACUC animal use protocol, 851. Mice exposed to sarin were housed and treated at MRI Global under an IACUC-approved protocol, 110804.01.002. All mice received food and water ad libitum during their acclimation at the animal facilities and during the course of the experiment.

### Study Design.

In this study, adult female C57BL/6 mice were given a dosing sequence that simulates chronic OP toxicity (LD_30_) in humans through the injection of 1.5 mg/kg of CBDP solubilized in saline and 0.5% DMSO 1 hour before the injection of sarin (Maxwell et al., 1987). Rodents, especially rats and mice, express excess amounts of carboxylesterase (CaE), an enzyme involved in the detoxification of OP agents (Maxwell et al., 1987). CaE irreversibly binds to sarin and thus reduces the amount of free sarin available to inhibit AChE. As a result, the presence of CaE causes the need for increased doses of sarin per body weight compared with the dose humans would need to produce toxicity (Maxwell et al., 1987). CBDP, a CaE inhibitor, can be used to lower the dose of sarin required in mice (Maxwell et al., 1987). As observed in previous studies, CBDP efficiently increased rodent sensitivity to OP poisoning, making its presence at low doses more representative of human exposure (Bošković, 1979). At a dose of 1.5mg/kg, CBDP did not inhibit AChE activity. Based on our previous experience in experiments with CBDP and sarin at comparable levels, seven mice per group were determined to provide satisfactory results in the experiments (Garrett et al., 2010, 2013). For the sarin experiments, the number of animals was increased to allow for 30% loss. The ∼LD_30_ dose of sarin was determined in preliminary experiments at MRI Global to be 0.04 mg/kg in these mice. This was followed 30 minutes later by a 20 mg/kg injection of Q-VD-OPh or Q-VE-OPh. Finally, four groups of mice also received 1.0 mg/kg diazepam 30 minutes after the sarin injection. Diazepam at 1 mg/kg was chosen as a dose following a small preliminary study suggesting that seizure activity was reduced but not completely stopped and is within the concentrations previously tested by other groups showing similar results (Harris et al., 1994; Tashma et al., 2001). The control mice were divided into nine different groups, i.e., controls with glycol+ethanol vehicle, Q-VD-OPh, Q-VE-OPh, CBDP + DMSO, CDBP + Q-VD-OPh, CBDP + Q-VE-OPh, CBDP + diazepam, CBDP + diazepam + QVD, and CBDP + diazepam + QVE. Six groups were designated for sarin treatment, i.e., CBDP + sarin (all groups receiving sarin received CBDP), sarin + QVD, sarin + QVE, sarin + diazepam, sarin + QVD + diazepam, and sarin + QVE + diazepam.

### Brain Region of Interest Collection.

A time point of 2 days post sarin/Q-VD-OPh-exposure was chosen based on previous studies suggesting that treatment with Q-VD-OPh provided a protective effect against seizure-induced brain ischemia and mortality at 24 hours post seizure, crossed the blood-brain barrier, and showed little to no toxicity in the animals (Braun et al., 2007). Futhermore, preliminary studies suggested that cytokines were elevated at 48 hours post exposure. Our goal was to allow time for seizures and a neuroinflammatory response to occur and to determine whether Q-VD-OPh would attenuate the response within a short 48-hour time frame. The 14-day time point was chosen as a midpoint to the 30-day elevation reported by Chapman et al. (2006) to determine whether prolonged seizure activity and neuroinflammation were affected by the single dose of Q-VD-OPh. At 2- and 14-day time points, mice were sacrificed and brains were collected for cytokine analysis. Brains were separated into left and right hemispheres and flash frozen in isopentane for 30 seconds for histologic analysis or flash frozen on dry ice for cytokine analysis. The frozen brain sections were thawed on ice, and the brain was cut into 1mm wide slabs using a slotted mouse brain block to allow access to the regions of interest as determined by comparison with a mouse brain atlas. The amygdala and hippocampus were placed in separate 0.5ml tubes and stored at –80°C until use.

### Cytokine Analysis.

For cytokine analysis, the regions of interest were homogenized in 150*µ*l 1XPBS and protein concentration was determined using the Bradford assay (Bradford, 1976). Homogenized regions of interest were used for cytokine analysis using the Mouse Cytokine Group 1 BioPlex 200 (Bio-Rad) assay kits. The BioPlex 200 was validated once monthly and calibrated during each run. A multiplexed standard curve was analyzed at the same time for each of the 23 analytes, allowing for quantification of each sample. Briefly, all samples were analyzed in duplicate on a BioPlex plate. Samples were incubated with the kit reagents in the 96-well plate and washed with appropriate buffers according to instructions from Bio-Rad. The BioPlex plate was analyzed in the BioPlex 200 plate reader at a low photomultiplier tube setting with bead count set to 50 and bead map set to 100 with a 60-second time limit. After each run, the plate was washed and incubated, and data was collected at a high photomultiplier tube setting to ensure capture of all analyte data from the plate. Data were normalized to the Bradford protein concentration and grouped according to the study group to which they belonged.

For the apoptosis assay, frontal cortex was used and processed in the same manner as for the amygdala and hippocampus. The BioPlex 200 was set up the same, and the BioPlex Apoptosis panel 3 was used to analyze active caspase 3, BCL-sL/Bak dimer, Mcl-1/Bak dimer, and Survivin. All other conditions were similar to the cytokine analysis. Samples were normalized to protein levels as determined by the Bradford method.

### Survival Analysis.

To determine the percent survival for mice that became moribund before the end of the study, the mice were observed every hour. A number of characteristics were identified for these mice, e.g., seizures, found dead, stretching, excessive scratching or grooming, rough hair coat, tremors, ataxic, hunched posture, yawning, or euthanized. The survival data record was then used to generate a survival curve. GraphPad Prism software (Boston, MA) was used to generate the survival curve and determine whether there was any significant difference. A log-rank (Mantel–Cox) test was recommended by GraphPad Prism for significant differences in survival curves. The *χ*^2^ was 3.012, the degrees of freedom (df) was 5, and the *P* value was 0.6981, indicating that the survival curves were not significantly different. This was further confirmed by a Log-rank test wherein the *χ*^2^ was 0.06113, the df was 1, and the *P* value was 0.8047. Once again, the summary was no significant trend at a *P* value set to 0.05. Finally, a Gehan–Breslow–Wilcoxon test was used to determine significance. The *χ*^2^ was 6.825, the df was 5, and the *P* value summary was not significant. Animals that expired prior to the end of the time point they were assigned to were not used for the cytokine or apoptosis analysis.

### Statistical Analysis.

All cytokine, apoptosis, and survival data were analyzed using STATISTICA software (Statsoft, v12) or Prism GraphPad software (Boston, MA). A multivariate ANOVA was used to evaluate statistical significance with *P* value < 0.05 considered significant. A Fisher’s least significant difference post hoc test was used to evaluate individual group differences. All values are reported as mean ± standard deviation. Survival analysis by GraphPad Prism was described in the preceding section, Survival Analysis.

## Results

### Survival Following Exposure to Sarin.

**Fig. 2.**
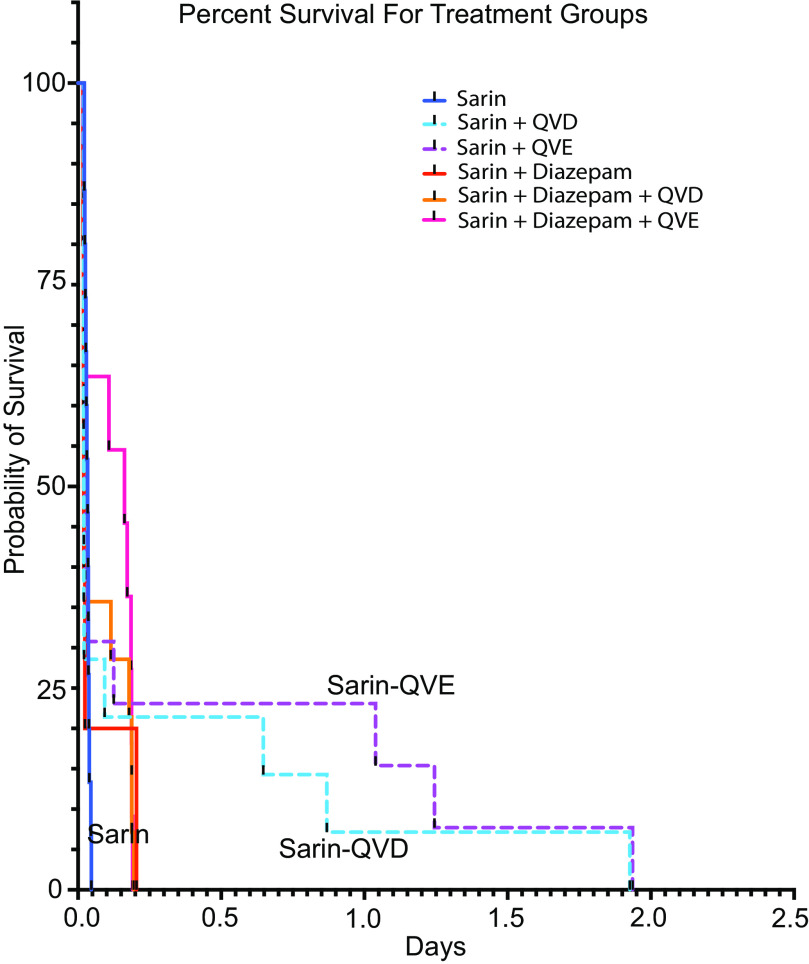
Percent survival curve for sarin-exposed mice and treatment groups. The graph is color coded and annotated to allow for easier identification of groups. Groups are sarin, *n* = 15; sarin-Q-VD-OPh, *n* = 14; sarin+Q-VE-OPh, *n* = 13; sarin+diazepam, *n* = 5; sarin+diazepam+Q-VD-OPh, *n* = 14; and sarin+diazepam+Q-VE-OPh, *n* = 11. Comparison of the survival curves was by Log-rank (Mantel–Cox) test, *χ*^2^ = 3.012l, df = 5, *P* value 0.6981; Log-rank test for trend *χ*^2^ = 0.06113, df = 1, *P* = 0.8047; Gehan–Breslow–Wilcoxon test *χ*^2^ = 6.825, df = 5, *P* = 0.234. The curves are not statistically different.

Signs of toxic exposure to sarin included immobility, seizures, tremors, hunched posture, lethargic, labored breathing, and not shredding enrichment material. These visible signs were observed within minutes of injection of sarin, peaking within 30 to 45 minutes but in some less severe cases lasting through the study, e.g., lethargic, hunched posture, and not shredding enrichment material. The concentration of sarin used in these experiments was determined to be at an LD_30-50_, and, as expected, there was significant mortality following sarin exposure. The time of injection and death as well as treatment group were recorded for each of these mice. Figure 2 suggests that mice treated with either Q-VD-OPh or Q-VE-OPh may have lived longer than mice not receiving these drugs. However, statistical analysis of this data showed no statistically significant difference in percent survival for those that died before the end of the experiment for any treatment (Fig. 2).

### The Presence of CBDP, Q-VD-OPh, and Q-VE-OPh Do Not Cause an Increase in Cytokines in the Amygdala or Hippocampus.

Before testing the effect of sarin on brain region specific neuroinflammation, it was first necessary to establish a baseline for the controls. We analyzed the amygdala lysates at 2- and 14-day time points for 23 cytokines using the BioPlex 200 assay. In the control amygdala at 2 days, we observed no significant effect of CBDP, Q-VD-OPh, or Q-VE-OPh on the cytokine levels (Supplemental Table 1A). In contrast to the 2-day control groups, at 14 days, the cytokine levels in 14 groups were below the level of detection for our instrument. These are indicated as OOR<. Since all sarin samples have CBDP, comparisons with CBDP and CBDP with Q-VD-OPh or Q-VE-OPh were used for the final analysis. Similar to the amygdala, baseline cytokine levels were first established in the hippocampus, followed by analysis of the sarin-exposed hippocampal regions. Analysis of the hippocampus lysates at the 2-day time point revealed very little difference in the control groups containing glycol or CBDP (Supplemental Table 2A). There was little difference in the cytokine levels at 14 days as well (Supplemental Table 2B).

**Fig. 3.**
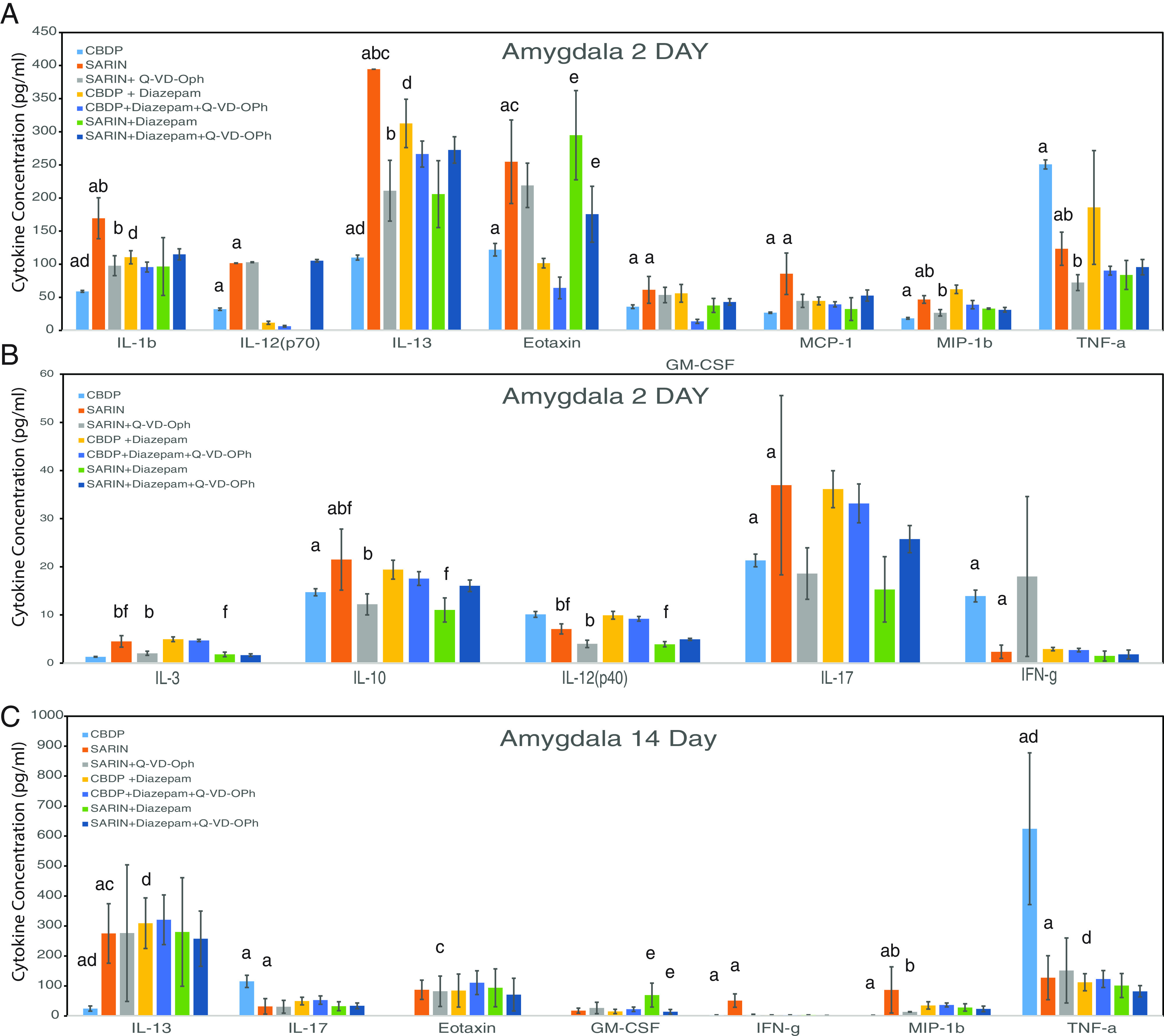
Cytokine analysis of amygdala at 2 and 14 days. Tissue lysates from the amygdala region of the mouse brain were analyzed using the Bio-Rad BioPlex instrument. Groups at 2 and 14 days are CBDP; sarin; sarin+Q-VD-OPh; CBDP + diazepam; CBDP + diazepam + Q-VD-OPh; and sarin + diazepam + Q-VD-OPh. Statistically significant differences (*P* < 0.05; described in Materials and Methods under statistics) are indicated by letters above the bars: (a) CBDP versus sarin; (b) sarin versus sarin + Q-VD; (c) 2-day sarin versus 14-day sarin; (d) CBDP versus CBDP + diazepam; (e) sarin + diazepam versus sarin + diazepam + Q-VD; (f) sarin versus sarin + diazepam. The data represent the mean ± SD for each group.

A previous report has indicated that anti-epileptics, i.e., midazolam, used to treat sarin or OP exposure can cause an increase in cytokines such as IL-1*β* (Chapman et al., 2006). Results from the diazepam experiments support this finding; in the amygdala at 2 days, IL-1*β* and IL-13 were both increased, and at 14 days IL-13 was still high while TNF-*α* was decreased compared with CBDP treatment alone (Fig. 3; Supplemental Table 3, A and B).

**Fig. 4.**
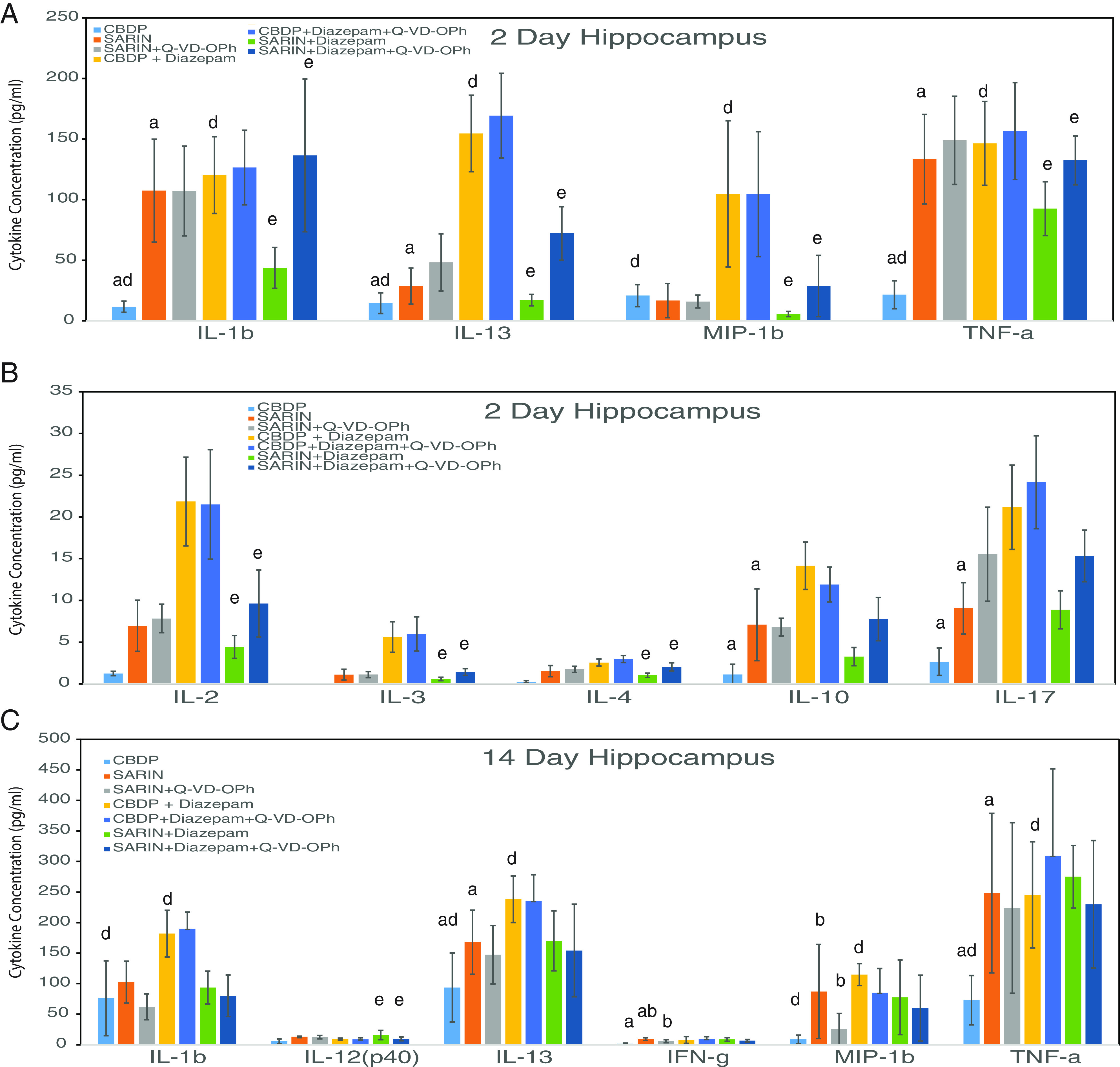
Cytokine analysis of hippocampus at 2 and 14 days. Tissue lysates from the hippocampal region of the mouse brain were analyzed using the Bio-Rad BioPlex instrument. Groups at 2 and 14 days are CBDP; sarin; sarin + Q-VD-OPh; CBDP + diazepam; CBDP + diazepam+Q-VD-OPh; and sarin + diazepam + Q-VD-OPh. Statistically significant differences (*P* < 0.05; described in Materials and Methods under statistics) are indicated by letters above the bars: (a) CBDP versus sarin; (b) sarin versus sarin + Q-VD; (d) CBDP versus CBDP + diazepam; (e) sarin + diazepam versus sarin + diazepam + Q-VD; (f) sarin versus sarin + diazepam. The data represent the mean ± SD for each group.

In hippocampus, the addition of diazepam appeared to cause a significant increase (*P* < 0.05) in four cytokines, IL-1*β*, IL-13, macrophage inflammatory protein (MIP)-1b, and TNF-*α* (Fig. 4; Supplemental Table 4A). On day 14 in the hippocampus, a similar significant difference (*P* < 0.05) was observed in the cytokine levels of IL-1*β*, IL-13, MiP-1b, and TNF-*α* (Fig. 4; Supplemental Table 4B).

### Sarin Elicits a Strong Cytokine Response in the Amygdala.

Having established a baseline, the first question to be answered was whether sarin would produce an inflammatory effect in specific brain regions and whether this effect would be limited to a few cytokines or to a wider range. Analysis of the amygdala lysates at 2 days comparing control (CBDP) and sarin-exposed tissues indicated there was a statistically significant change (*P* < 0.05), i.e., an increase in 9 and a decrease in 2 of the cytokines (i.e., TNF-*α* and interferon-*γ*) (Fig. 3; Supplemental Table 3A). At 14 days, there were fewer identified differences: only IL-13, molecularly imprinted polymers-1b, IL-17, IFN-*γ*, and TNF-*α*, the latter two of which were lower in the sarin group than in the CBDP controls (Fig. 3; Supplemental Table 3B). When the levels of the amygdala cytokines at 2 and 14 days were compared for mice exposed to sarin, two cytokines were found to be significantly different (*P* < 0.05), i.e., IL-13 and eotaxin, both of which decreased (Fig. 3, A and B; Supplemental Table 3, A and B).

### Sarin Elicits a Strong Cytokine Response in the Hippocampus.

In the hippocampus of animals exposed to sarin, an increase was observed in far fewer, i.e., five, of the cytokines at the 2-day time point (Fig. 4; Supplemental Table 4A), and on day 14 after sarin exposure, there appeared to be only three of the cytokines affected: IL-13, interferon (IFN)-*γ*, and TNF-*α* (Fig. 4; Supplemental Table 4B). Comparison of the 2- and 14-day sarin-sensitive cytokines showed no significant difference in the cytokine levels between the two days (Supplemental Table 2, A and B).

### Q-VD-OPh Attenuates the Inflammatory Response at 2 Days.

The main objective of the project was to determine whether the inflammatory response initiated by sarin could be attenuated in some way by treatment with the broad-spectrum caspase inhibitor Q-VD-OPh. At the 2-day time point for amygdala, seven cytokines were found to be significantly decreased (*P* < 0.05) following treatment with Q-VD-OPh, i.e., IL-1*β*, IL-13, MIP-1b, IL-3, IL-10, IL-12(p40), and TNF-*α* (Fig. 3A; Supplemental Table 3A). In the amygdala at 14 days, MIP-1b showed a significant decrease (*P* < 0.05) (Fig. 3B; Supplemental Table 3B). Testing Q-VE-OPh for the same showed a significant decrease in TNF-*α* at 2 days and a significant increase in IL-13 at 14 days (*P* < 0.05), though both differences appeared to be due to the presence of sarin (Supplemental Table 1, A and B).

In hippocampus, at 2 days there did not appear to be an effect of Q-VD-OPh on any of the cytokines (Fig. 4, A and B; Supplemental Tables 2 and 4, A and B). At 14 days, Q-VD-OPh caused a decrease in IFN-*γ* (*P* < 0.05). Q-VE-OPh did not appear to have an effect on the sarin-induced cytokine levels at 2 or 14 days in the hippocampus (Supplemental Table 2, A and B). Sarin did cause an increase in hippocampal IL-1*β*, IL-13, and TNF-*α* in Q-VE-OPh-treated animals at 2 days (Supplemental Table 2A). At 14 days, only IL-13 and TNF-*α* remained significantly increased with the addition of sarin (*P* < 0.05) (Supplemental Table 2B).

### Diazepam Does Not Provide Additive Capacity to Q-VD-OPh.

Standard of care for sarin exposure is to administer an antiepileptic drug, e.g., diazepam (Tattersall 2009), though recently midazolam has become the standard of care as proposed by Reddy and Reddy (2015). To test its effectiveness against sarin, diazepam was injected 30 minutes after treatment with Q-VD-OPh. There was an apparent effect of diazepam on three cytokines in the amgdala of sarin-exposed animals at 2 days, i.e., IL-3, IL-10, and IL-12(p40) (Fig. 3, A and B; Supplemental Table 3, A and B). Diazepam in conjunction with Q-VD-OPh was found to have a statistically significant impact on eotaxin at 2 days and granulocyte-macrophage colony-stimulating factor at 14 days in the amygdala (Fig. 3, A and B; Supplemental Table 3, A and B). In contrast to the amygdala, the addition of diazepam to Q-VD-OPh appeared to be markedly different in the hippocampus. In the hippocampus, when mice exposed to sarin were treated with diazepam or diazepam + Q-VD-OPh, instead of reducing the inflammatory response, an increase inflammation in seven inflammatory cytokines in the Q-VD-OPh-containing group was observed at 2 days (Fig. 4A; Supplemental Table 4A). In contrast, there was only one statistically significant decrease in the cytokine IL-12 (p40) at 14 days in the hippocampus (Supplemental Table 4B). Q-VE-OPh + diazepam did not have an effect on the inflammatory response in amygdala at 2 or 14 days (Supplemental Table 1, A and B). In contrast, at 2 days in the hippocampus, the exposure to sarin caused a decrease in IL-1*β*, MIP-1b, and TNF-*α*, while at 14 days there was no difference in the cytokine levels (Supplemental Table 2, A and B). The results suggest that diazepam did have an influence on some of the cytokines after treatment of OP exposure with Q-VD-OPh in both tissues.

### Assay for Apoptosis in the Frontal Cortex.

**Fig. 5.**
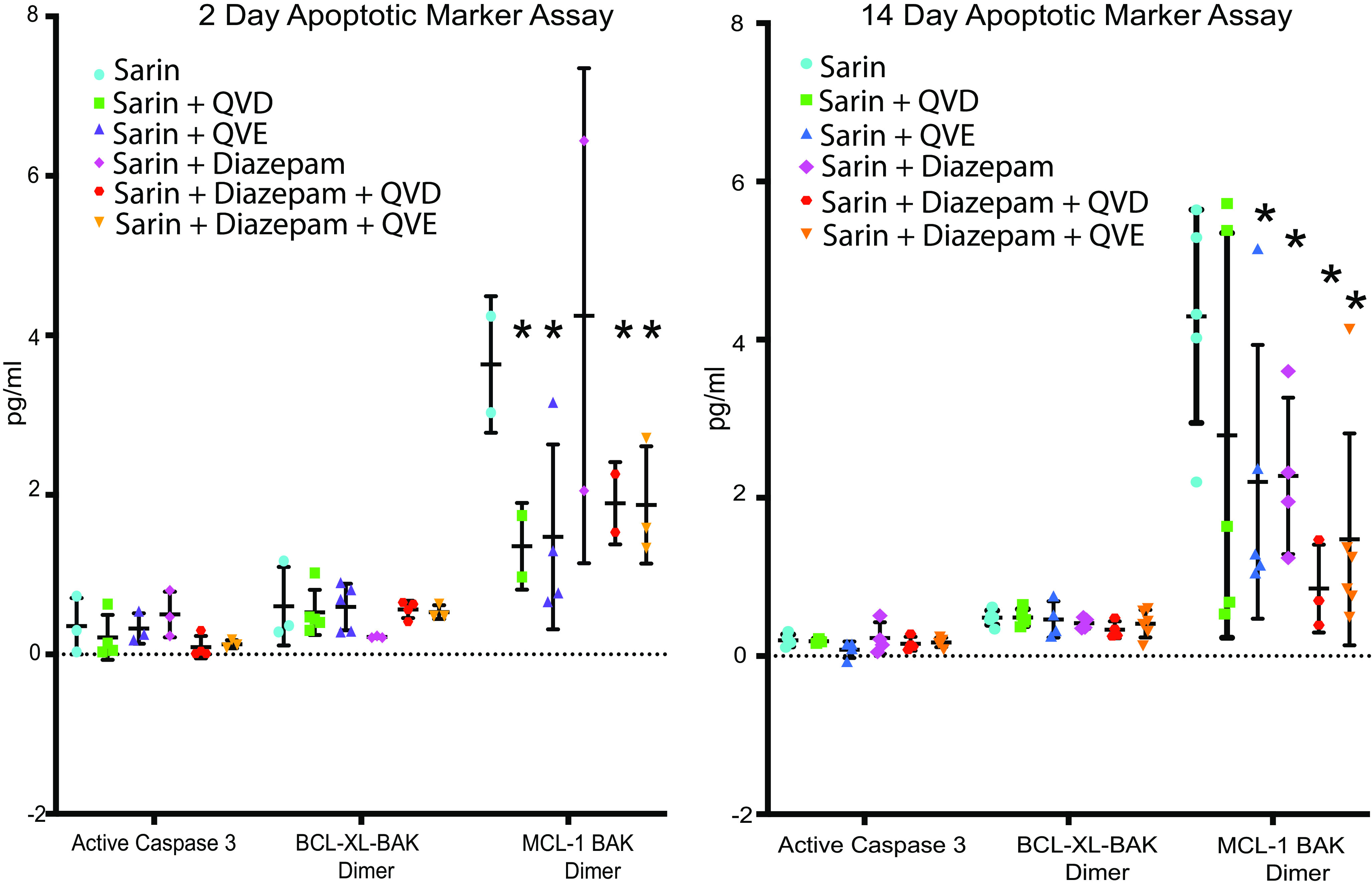
Multiplex apoptotic marker evaluation. Frontal cortex tissue was analyzed using the Bio-Rad BioPlex instrument. Groups are sarin; sarin+Q-VD-OPh; sarin+Q-VE-OPh; sarin+diazepam; sarin+diazepam+Q-VD-OPh; and sarin+diazepam+Q-VE-OPh. The assays are for Caspase 3, BCL-XL-BAK Dimer, and MCL-1 BAK Dimer. Survivin is not shown. Two-way ANOVA was used to analyze the data with *P* < 0.05 for all statistically significant values (*) compared with sarin. The data represent the mean ± SD for each group.

To test for the presence of sarin-induced apoptosis, we used tissue from the frontal cortex since the amount of amygdala or hippocampal tissue was limited by their use in previous experiments. In the multiplexed apoptosis assay, four indicators of apoptosis were analyzed, i.e., active caspase 3, BCL-XL-BAK dimer, MCL-1 BAK dimer, and survivin. Survivin data are not shown as the levels were mostly below detectable limits. At 2 and 14 days in the frontal cortex, only MCL-1 BAK dimer showed significantly elevated values (Fig. 5A). At 14 days, the data indicates that sarin was significantly different from all other treatment groups except sarin-QVD (Fig. 5B) (*P* < 0.05).

## Discussion

Inflammatory responses in the peripheral and central nervous systems play important roles in the development of pathologies in response to toxic insults such as OP. Modulation of the inflammatory response is representative of the balance between proinflammatory cytokines and anti-inflammatory cytokines (Vexler et al., 2006). In this study, we evaluated 23 cytokines and chemokines in two specific brain regions, i.e., the amygdala and the hippocampus, following exposure to sarin. This is important as most studies focus on only a few cytokines or chemokines due to the high cost of individual ELISAs for each analyte, the limited amount of sample available for each ELISA, and the sensitivity of the particular ELISA. The introduction of the Luminex-style bead-based system that can analyze multiple analytes, e.g., 23 to 100, in a small sample volume, i.e., 50 *μ*l, provides a unique approach toward moving past these limitations. Evidence provided here indicates that all 23 cytokines were found in both the amygdala and hippocampus of the mouse brain. While not all of these were sensitive to the presence of sarin, this larger collection of cytokines provides a better understanding of a broader range of inflammation that may play a role in the toxic short- and long-term effects of OPs in the brain.

The first phase of this study was to determine what effect sarin would have on the inflammatory response in these two regions. Previous studies have shown sarin and other OPs to increase levels of cytokines such as IL-1*β*, TNF-*α*, and PGE2 in the hippocampus and cortex (McDonough and Shih 1993, Chapman et al., 2006, Burguillos et al., 2011, Garrett et al., 2013). In one set of experiments, the inflammatory markers PGE2, IL-1*β*, IL-6, and TNF-*α* were elevated rapidly, decreased at 6 days, followed by a secondary increase through 30 days following at least 30 minutes of seizure activity (Chapman et al., 2006). This was proposed to be due to a mechanism whereby microglia were activated by seizure activity following exposure to soman, causing the prolonged response (Zimmer et al., 1997). In another study, proinflammatory cytokine genes such as IL-1*β*, IL-6, and TNF-*α* were all found to be increased in response to sarin or DFP exposure in the amygdala, hippocampus, cortex, or thalamus (Henderson et al., 2002; Spradling et al., 2011; Li et al., 2015). In those studies, the timing was within hours of the injection of sarin lasting up to the end of the experiment at 24 hours. The results from the current study were unambiguous; i.e., sarin had a significant effect on multiple cytokines analyzed in both the amygdala and hippocampus at 2 and 14 days compared with the CBDP-only controls. While this is not unexpected, the additional information on cytokines other than the standard set always studied was noteworthy. Of interest, in our study, TNF-*α* was mostly decreased in the presence of sarin. We did not test mRNA levels, and the experiment ran for 2 or 14 days. The increase in proinflammatory cytokines in both the amygdala and hippocampus strongly suggests that sarin has an influence on the inflammatory pathways in the brain. It should be noted that there is nearly always a background level of various cytokines in the brain as well as other tissues in the body. In the control amygdala tissue at 14 days, 14 cytokines were below the level of detection for the BioPlex instrument to read. In the hippocampus, only six cytokines were below the level of detection at 14 days. Occasional loss of data is expected, but the randomization of the sample placement on the plates coupled with other samples with positive output on the same plate and the fact that all samples were run in duplicate suggests that these were not misreads or an error in the instrument or sample preparation. The most probable explanation is that these readings could be due to the acclimation time to the new environment after shipping during which stress levels became less and thus the baseline inflammatory responses were low. However, although there is a lack of controls for these cytokines at 14 days, the effect of sarin or diazepam should not be discounted. We propose that there is a large biologically relevant increase in the cytokine levels due to sarin or diazepam even though a statistical measurement cannot be determined.

In this study, we found no significant change in the levels of cytokines in the control amygdala and hippocampus of CBDP-treated animals at 2 or 14 days (see Supplemental Tables 1 and 2). There are no reports in the literature suggesting that CBDP may have an effect on cytokine levels or inflammation in the brain or other tissues, and our results support that.

Our next goal was to determine whether Q-VD-OPh alone had any effect on cytokines in the two regions of the brain. Q-VD-OPh is a pan caspase inhibitor with a wide spectrum and is postulated to have an indirect effect on the immune system via the caspase 1 pathway (Pan et al., 2016). Q-VE-OPh is 20 times less effective as a caspase inhibitor than Q-VD-OPh, making it a good negative control for apoptosis, and, thus, it should not have had an effect on cytokine levels (Southerland et al., 2010). In this study, we did not observe changes in cytokines at 2- and 14-day time points in the control amygdala due to treatment with Q-VD-OPh or Q-VE-OPh compared with CBDP. However, when sarin-exposed mice were treated with Q-VD-OPh, the effect was a reduction in seven of the cytokine levels in the amygdala. In contrast, at 14 days in the amygdala, the only difference in any of the cytokine levels following treatment with Q-VD-OPh was in MIP-1b. This strongly suggests that Q-VD-OPh can alter the inflammation induced by sarin up to 2 days following sarin exposure and subsequent treatment. By comparing the Q-VD-OPh results at 2 and 14 days, we found that the Q-VD-OPh sensitive neuroinflammatory response was transient and returned by the 14th day of the study. This was not unexpected, as there was only one treatment with Q-VD-OPh, and sarin/OP effects, especially when seizure activity is present, tend to be long term (Casida and Quistad 2004, Chapman et al., 2006). In the hippocampus, IFN-*γ* was the only cytokine reduced by Q-VD-OPh, and it was at the 14-day time point. This could indicate either a lag in time for the initial response to Q-VD-OPh compared with the amygdala response, that the reduction had already occurred and was returning to previous levels, or that the Q-VD-OPh did not have a strong effect in the hippocampus. Q-VE-OPh was not able to attenuate the cytokine levels induced by sarin.

Caspase 1 is involved in the cleavage of immune proteins that trigger a strong neutrophil response, suggesting that a caspase inhibitor could have such an effect (Alphonse et al., 2021). In another recent study, a caspase 1 specific irreversible inhibitor (YVAD) was shown to reduce inflammatory cytokine levels for IL-1*β*, IL-6, and TNF-*α* in a macrophage model system (Pan et al., 2016). Previous studies on inflammation and Q-VD-OPh also suggest that it has the capacity to inhibit or attenuate microglial activation and that its anti-apoptotic effects could mediate inflammatory responses (Burguillos et al., 2011; Keoni and Brown, 2015). Our results provide evidence supporting these studies. While Q-VD-OPh inhibition is not specific to any one caspase, its ability to regulate the neuroinflammatory response in these mice provides support for the future study of caspase inhibitors in attenuation of OP-induced neuroinflammation.

### Diazepam Causes an Inflammatory Response.

Previous studies have shown an effect for anti-epileptics, i.e., midazolam, to cause an increase in the neuroinflammatory response by increasing cytokine production, specifically IL-1*β* (Chapman et al., 2006). In the current study, we show that diazepam does cause a limited increase in specific cytokine levels, i.e., Il-1*β*, and IL-13 in amygdala at 2 days. By 14 days, only IL-13 was affected by diazepam, though TNF-*α* appeared to be reduced. In the hippocampus, the effect was more pronounced with four cytokines increased at 2 days and the remaining increased at the 14-day time point. This suggests that anti-epileptics are not without some contribution to neuroinflammation, though further study is warranted.

### Apoptotic Markers Indicate a Prolonged Effect of Q-VD-OPh.

The frontal cortex was used to test for four apoptotic markers, and, while all were present, only the MCL-1 BAK dimer gave statistically and potentially biologically relevant data. MCL-1 is involved in apoptosis as an anti-apoptotic member of a larger family of prosurvival proteins that bind to BAK to form a dimer preventing apoptosis (Van Cruchten and Van Den Broeck, 2002; Willis et al., 2005; Adams, 2007). This binding is through an aspartic acid residue, and we speculate that the aspartic acid in Q-VD-OPh may compete for this site and disrupt the dimerization (Willis et al., 2005).

### Does Q-VD-OPh Have a Positive Effect on the Inflammatory Response?

The type of cytokines sensitive to sarin and Q-VD-OPh appear to be proinflammatory, e.g., IL-1*β*, IL-10, IL-13, or chemokines, e.g., Rantes, MCP-1, and MIP-1b. MIP-1b has been identified with bacterial lipopolysaccharide exposure and is one of a group of chemokines responsible for attracting CD4+ lymphocytes (Taub et al., 1993) Their presence at 2 days suggests that the inflammatory process is ongoing in the mice exposed to sarin. In the mice treated with a single dose of Q-VD-OPh at 30 minutes after sarin exposure, the evidence suggests that it does still have an effect at 2 days. While not all the cytokine levels returned to normal or CBDP level, those that were lowered showed that Q-VD-OPh may offer protection if given in an extended dose. In contrast, some cytokines that are insensitive to sarin and to Q-VD-OPh at 2 days may have been impacted earlier or may be affected later in an extended inflammatory response. This delayed neuropathic response is considered to be one of the confounding problems following early treatment and survival of OP-exposure patients (Ehrich and Jortner, 2001). This further suggests the importance of knowing more about the inflammatory response ongoing in these sarin-exposed animals. We have provided an expanded view of the inflammatory response that should help play a role in the future of treatment, diagnosis, the pathways responsible for this release, and their interactions with other cellular systems that regulate the neuroinflammatory response.

## Abbreviations

AChE: acetylcholinesterase
CaE: carboxylesterase
CBDP: 2-(o-cresyl)-4H-1:3:2-benzodioxaphosphorin-2-oxide
IL: interleukin
IFN: interferon
MIP: macrophage inflammatory protein
OP: organophosphate
Q-VD-OPh: quinolyl-valyl-O-methylaspartyl-[-2,6-difluorophenoxy]-methyl ketone
Q-VE-OPh: quinolyl-valyl-O-methylglutamyl-[-2,6-difluorophenoxy]-methyl ketone
PGE2: prostaglandin E2
TNF: tumor necrosis factor
